# Incorporation of Real-Time PCR into Routine Public Health Surveillance of Culture Negative Bacterial Meningitis in São Paulo, Brazil

**DOI:** 10.1371/journal.pone.0020675

**Published:** 2011-06-22

**Authors:** Claudio T. Sacchi, Lucila O. Fukasawa, Maria G. Gonçalves, Maristela M. Salgado, Kathleen A. Shutt, Telma R. Carvalhanas, Ana F. Ribeiro, Brigina Kemp, Maria C. O. Gorla, Ricardo K. Albernaz, Eneida G. L. Marques, Angela Cruciano, Eliseu A. Waldman, M. Cristina C Brandileone, Lee H. Harrison, São Paulo RT-PCR Surveillance Project Team

**Affiliations:** 1 Division of Medical Biology, Department of Immunology, Instituto Adolfo Lutz, São Paulo, Brazil; 2 Infectious Diseases Epidemiology Research Unit, University of Pittsburgh Graduate School of Public Health and School of Medicine, Pittsburgh, Pennsylvania, United States of America; 3 Center for Epidemiologic Surveillance, São Paulo, Brazil; 4 Center for Epidemiologic Surveillance, Campinas, Brazil; 5 Division of Medical Biology, Department of Bacteriology, Instituto Adolfo Lutz, São Paulo, Brazil; 6 Bacteriology Area, Department of Medical Biology, Instituto Adolfo Lutz Regional Laboratory of Campinas, Campinas, Brazil; 7 Department of International Health, Johns Hopkins Bloomberg School of Public Health, Baltimore, Maryland, United States of America; 8 Faculdade de Saúde Pública, Universidade de São Paulo, São Paulo, Brazil; Naval Research Laboratory, United States of America

## Abstract

Real-time (RT)-PCR increases diagnostic yield for bacterial meningitis and is ideal for incorporation into routine surveillance in a developing country. We validated a multiplex RT-PCR assay for *Streptococcus pneumoniae, Neisseria meningitidis*, and *Haemophilus influenzae* in Brazil. Risk factors for being culture-negative, RT-PCR positive were determined. The sensitivity of RT-PCR in cerebrospinal fluid (CSF) was 100% (95% confidence limits, 96.0%–100%) for *N. meningitidis*, 97.8% (85.5%–99.9%) for *S. pneumoniae*, and 66.7% (9.4%–99.2%) for *H. influenzae*. Specificity ranged from 98.9% to 100%. Addition of RT-PCR to routine microbiologic methods increased the yield for detection of *S. pneumoniae*, *N. meningitidis*, and *H. influenzae* cases by 52%, 85%, and 20%, respectively. The main risk factor for being culture negative and RT-PCR positive was presence of antibiotic in CSF (odds ratio 12.2, 95% CI 5.9-25.0). RT-PCR using CSF was highly sensitive and specific and substantially added to measures of meningitis disease burden when incorporated into routine public health surveillance in Brazil.

## Introduction

Bacterial meningitis is a serious and often fatal infection. *Neisseria meningitidis, Streptococcus pneumoniae,* and *Haemophilus influenzae* type b (Hib) account for the vast majority of bacterial meningitis cases outside the neonatal period. Understanding the burden of bacterial meningitis is important because of recent advances in vaccines for these infections. Hib conjugate vaccines have led to the near disappearance of invasive Hib disease in Brazil and elsewhere [1]. Pneumococcal conjugate vaccine has had a huge impact on the incidence of invasive pneumococcal disease in the United States and is being incorporated into the routine pediatric immunization schedule in Brazil, as is serogroup C meningococcal conjugate vaccine [2], [3].

In many developing countries, surveillance for bacterial meningitis is hampered by limited use of bacterial culture and a high frequency of negative cultures [4]. Availability of over-the-counter antibiotics, administration of antibiotics before performance of lumbar puncture, lack of microbiology resources for bacterial culture, and variable quality of microbiology services are among the reasons for culture negativity. This problem leads to an underestimate of disease burden and assessments of the potential impact of vaccination. Non-culture methods, such as real-time (RT)-PCR, can increase the diagnostic yield for bacterial meningitis in both developed and developing countries [4], [5], [6], [7], [8], .

Much of what is known about the incidence of bacterial meningitis in Brazil is from the Brazilian Reportable Diseases Surveillance System (Sistema de Informação de Agravos de Notificação [SINAN]), which conducts public health surveillance for notifiable diseases. An annual average of 28,000 cases of bacterial meningitis is reported nationwide, which likely underestimates disease burden because SINAN is a passive system. About half of reported pyogenic meningitis cases are culture negative, suggesting that use of RT-PCR could enhance measurement of disease burden. The purpose of this study was to validate RT-PCR when incorporated into routine public health surveillance and identify factors associated with being culture negative, RT-PCR positive in São Paulo.

## Methods

### Institutional review board (IRB) approvals

The study was approved by the Instituto Adolfo Lutz (IAL) and University of Pittsburgh IRBs, as well as the Comissão Nacional de Ética em Pesquisa, the Brazilian national IRB.

### Data sources

We included nine hospitals in the city of São Paulo (Instituto de Infectologia Emílio Ribas, Casa de Saúde Santa Marcelina, Hospital Estadual do Grajaú, Hospital da Santa Casa de Misericórdia de São Paulo, Hospital Municipal Infantil Menino Jesus, Hospital São Paulo, Hospital Estadual Regional Sul, Conjunto Hospitalar do Mandaqui, and Hospital Municipal do Tatuapé) and three in nearby Campinas (Hospital das Clínicas-Unicamp, Hospital Mario Gatti, and Hospital Celso Pierro), selected because they are centers for treatment of bacterial meningitis, report through SINAN, and send specimens for performance of counter immunoelectrophoresis for *N. meningitidis* and Hib at IAL, a São Paulo State reference laboratory, or to IAL Regional Laboratory of Campinas. IAL serves as the Brazilian National Reference Laboratory for Bacterial Meningitis. Eleven of the 12 hospitals conduct active surveillance for meningitis.

SINAN was queried for patients from these facilities for May 4, 2007 through March 31, 2009. Two non-mutually-exclusive groups of patients were included: those with blood or cerebrospinal fluid (CSF) culture positive for one of the three study organisms and patients whose CSF had ≥100 leukocytes/mm^3^ and ≥60% neutrophils, regardless of culture results. Patients in the culture positive group were used in the analyses of the sensitivity of RT-PCR, regardless of whether they met the CSF criteria. Patients who were culture negative but met the CSF criteria were used to determine the additional yield of RT-PCR. Other patients with suspected meningitis who did not meet these criteria but who had CSF submitted to IAL were used for the survival analyses described in the data analysis section.

Information gathered from SINAN included results of blood and CSF culture, demographic variables, and clinical and laboratory variables. Medical record reviews were conducted to collect information that was missing from SINAN. CSF and serum specimens were sent to IAL for RT-PCR testing.

Culture results were obtained from SINAN, which were confirmed during site visits to each of the hospital laboratories. Some isolates were also sent to IAL for confirmation; those results were added to the database if missing from SINAN.

### Laboratory assays

A multiplex RT-PCR assay was used to identify patients with bacterial meningitis caused by the three study organisms. The assay was developed and performed at IAL and composed of three sets of primers and a probe targeting 1), meningococcal capsular transport gene *ctrA* [11], 2) pneumococcal autolysin gene *lytA* [12], and 3) *H. influenzae* polysaccharide capsular expression gene *bexA*, which detects *H. influenzae* serotypes a, b, c, and d, but not e and f. [4].

The laboratory sensitivity of the assay was assessed with 55 isolates of *N. meningitidis*, 54 *H. influenzae*, and 66 *S. pneumoniae*. For laboratory specificity, 125 bacterial and fungal isolates representing other species were used to validate the three individual RT-PCR assays (*ctrA, lytA* and *bexA*), and also the multiplex RT-PCR assay: *Acinetobacter baumannii, Cryptococcus sp, Coccidioides immitis, Chlamydia pneumoniae, Chlamydia trachomatis, Corynebacterium ulcerans, Corynebacterium diphtheria, Haemophilus parainfluenzae, Haemophilus influenzae* biogroup aegyptius, *Enterobacter cloaceae, Enterococcus faecalis, Histoplasma capsulatum, Klebsiella pneumoniae, Legionella sp, Leptospira interrogans, Moraxella catarrhalis, Moraxella genitalium, Mycobacterium tuberculosis, Nocardia asteroides, Pseudomonas aeruginosa, Paracoccidioides brasiliensis, Pneumocystis carinii, Streptococcus agalactiae, Streptococcus mitis, Streptococcus pyogenes, Neisseria lactamica, Neisseria sicca, Neisseria subflava, Salmonella sp, Streptococcus viridians,* and *Listeria monocytogenes*.

The lower limit of detection (LLD) was determined using extracted DNA from one isolate each of *N. meningitidis, H. influenzae* and *S. pneumoniae*. DNA concentration was adjusted to 100 ng/µL from which 10 fold serial dilutions (10^−1^ to 10^−9^) were made in PCR-grade water. The LLD for the individual and multiplex assays were determined to be the dilution that yielded a Ct value ≤39.

DNA extraction was performed using QIAGEN QIAamp DNA Blood mini kit (QIAGEN, Valencia, CA) as described by the manufacturer except that ideally an aliquot of 500 μL of CSF or 200 μL of serum was processed and the DNA was eluted in 50 μL or 100 μL of TE buffer, respectively. However, specimens with smaller available volumes, representing around 40% of specimens, were also included. 200 µL of PCR-grade water was included as an extraction-negative control.

RT-PCR reactions were prepared in a separate area in which no DNA or bacteria were handled and in a biosafety cabinet cleaned with 1% sodium hypochlorite and exposed to UV light for at least 20 minutes. Final reactions were prepared in 96 well plates that included three separate positive controls, one for each of the target organisms: strain N.24/75 serogroup A *N. meningitidis*, strain ST.782/06 serotype 3 *S. pneumoniae*, and H.18/06 Hib. These controls were prepared in 0.01 M PBS pH 7.2 with 0.02% sodium azide, heated to 56°C for 30 minutes, and maintained at −20°C. The extracted water and four reactions without any DNA template were also included in each plate as negative controls.

The assays were carried out in a final 25 µl reaction volume and were performed using TaqMan Universal Master Mix (Applied Biosystems, Foster City, CA), with 5 µl of sample extracted DNA. Forward primer, reverse primer, and probe for each gene target were used in concentrations of, respectively, 300, 900, and 100 nM for *ctrA*; 300, 600, and 100 nM for *lytA*; and 300, 300, and 100 nM for *bexA*. The *ctrA*, *lytA*, and *bexA* probes were labeled at the 5′ end with FAM, VIC, and NED, respectively.

All reactions were run in duplicate and the assays were performed using an Applied Biosystems 7300 Real-Time PCR System (Applied Biosystems) using the following cycling parameters: 50°C for 2 min, 95°C for 10 min, followed by 45 cycles of 95°C for 15 s and 60°C for 1 min. Extension of 55°C for 1 min was used for single RT-PCR genogrouping assay for serogroups B, W135, and Y. A positive result was defined as a cycle threshold (Ct) value below 39, an inconclusive result as a Ct value of 40 or 41, and a negative result as a Ct value of zero or >41. All inconclusive results and inconsistent replicates were repeated. Other than Ct values, the positive reactions were confirmed for each probe by the increase in fluorescence during the RT-PCR reaction. To determine the influence of RT-PCR Ct cut-off value on assay sensitivity, analysis using Ct values ≤35 as a second definition for positivity was used for all culture positive samples.

For further evaluation of culture-negative, RT-PCR-positive specimens, confirmation using a second gene was performed using RT-PCR assays targeting group-specific genes of *N. meningitidis* [11], *ply* from *S. pneumoniae* [4], or type-specific genes for *H. influenzae* [13]. The meningococcal group-specific assay was also used to determine meningococcal group.

CSF specimens with sufficient volume were tested for the presence of antibiotic [6], [14]. A 5 mm blank filter paper disc was soaked with 15 µL of CSF and placed on Mueller Hinton agar plate with a lawn of the pansensitive bacterium *Kocuria rhizophila* (ATCC 9341). The plate was incubated overnight at 37°C and zone of inhibition around the disc was measured at 24 hours. Any zone was considered positive for presence of antibiotic. A disc containing 0.015 U of penicillin was used as a positive control and a blank disk was used as a negative control.

### Data analysis

The sensitivity of RT-PCR for each pathogen was calculated as the proportion of culture (blood and/or CSF) positive specimens that had a positive result by RT- PCR for the same pathogen. Specificity was calculated using two approaches. In the first, as the proportion of all patients who were culture positive for one of the other two pathogens that were RT-PCR negative for the pathogen in question (specificity method 1). Patients who were culture positive for organisms other than *N. meningitidis, S. pneumoniae* or *H. influenzae* (e.g., *Escherichia coli*) were excluded. The presumption was that specimens that are, for example, culture positive for *N. meningitidis* or *S. pneumoniae* should be negative by RT-PCR for *H. influenzae*. In the second, culture-negative specimens that met the CSF criteria of ≥100 leukocytes/mm^3^ and ≥60% neutrophils were also included (specificity method 2, unadjusted. To determine whether RT-PCR positive, culture-negative specimens represented true RT-PCR positives (because of false negative culture results), these specimens were tested by PCR for a second gene target as indicated above. Specimens that were positive for the second target were interpreted as being true positives and therefore removed from the specificity calculation (specificity method 2, adjusted).

For the analyses of percent of patients with positive RT-PCR assay by CSF WBC count and CSF percent neutrophils, patients who were culture negative and who met the CSF criteria were included. CSF specimens that were culture negative or culture unknown that were submitted for routine testing for suspected bacterial meningitis, regardless of CSF profile, were also included. Survival analyses were performed to determine the proportion of RT-PCR positive samples that would be detected and the proportion of samples that would be tested at any specific WBC value.

To investigate factors associated with being culture-negative, cases were defined as patients who met the CSF criteria above and were culture negative and RT-PCR positive. Controls were patients who had positive CSF and/or blood cultures. Univariate logistic regression was done in SAS (version 9.2, SAS Institute) to identify risk factors. Transformations were performed on continuous factors that were not approximately linear to facilitate inclusion in the model. Factors with a p-value <0.1 were eligible for inclusion in multivariable logistic regression analysis. A backward logistic regression model with a stay criterion of 0.05 was run to identify independent risk factors for being culture negative. Factors remaining in the final model were checked for colinearity.

## Results

### Laboratory validation of RT-PCR assay

All *N. meningitidis, H. influenzae* and *S. pneumoniae* used in the evaluation of sensitivity of the RT-PCR assays were positive in the species-specific assay targeting the *ctrA, bexA* and *lytA* genes, respectively (laboratory sensitivity of 100%). DNA from strains representing other species gave negative results in all assays (laboratory specificity of 100%). The LLD for the individual and multiplex RT-PCR assays was 20 fg of genomic DNA.

### Validation of RT-PCR assay on clinical specimens

**Figure 1:**
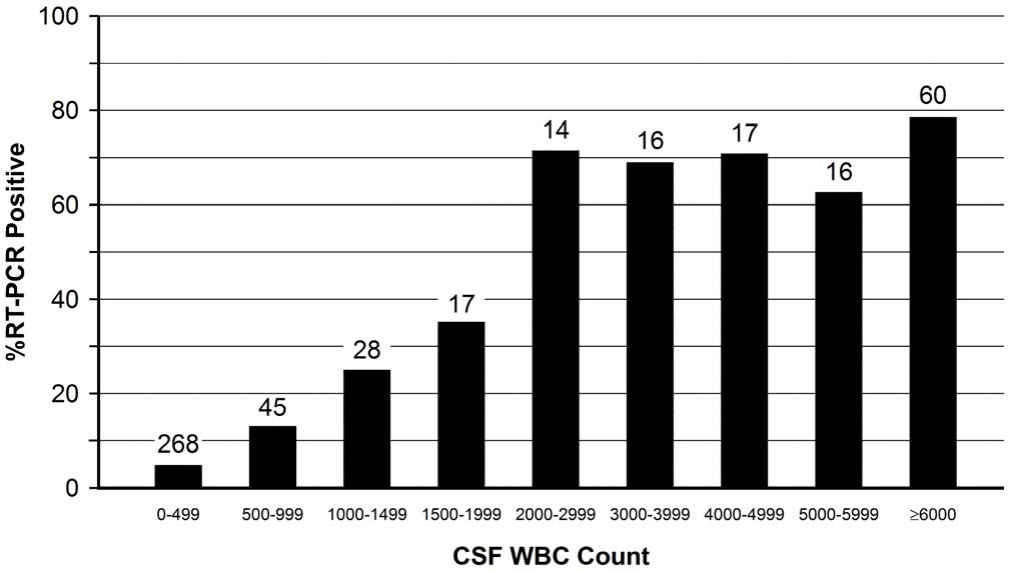
Proportion of culture-negative and culture-unknown 481 CSF specimens that were RT-PCR positive, by cerebrospinal fluid (CSF) white blood cell (WBC) count (leukocytes/mm^3^). Numbers above bars represent the number of specimens tested. Includes 188 patients with unknown culture results.

**Figure 2:**
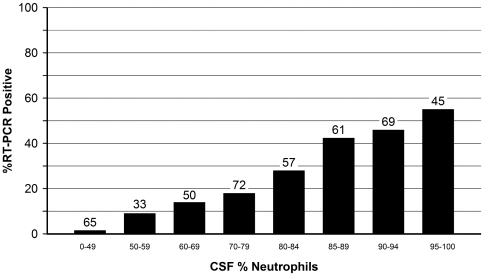
Proportion of culture-negative and culture-unknown 452 CSF specimens that were RT-PCR positive, by cerebrospinal fluid (CSF) percent neutrophils. Numbers above bars represent the number of specimens tested. Includes 188 patients with unknown culture results.

**Figure 3:**
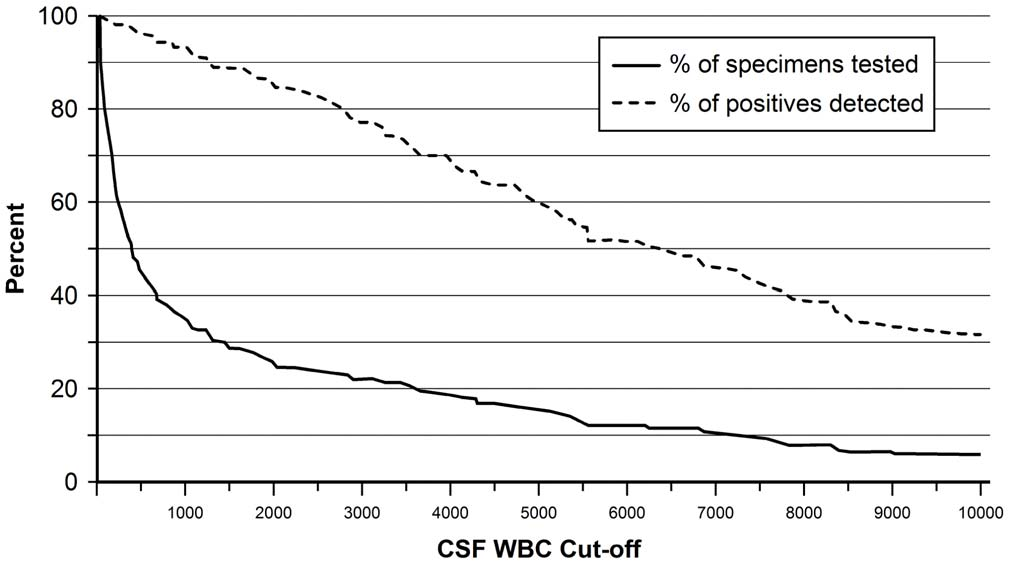
Relationship between minimum CSF WBC count used as a cut-off to determine what specimens are tested by RT-PCR and 1) the proportion of the total RT-PCR positives (n = 122) that are identified (dashed line) and 2) the proportion of all specimens (n = 481) that would be tested (solid line). For example, if only those specimens with a CSF WBC of at least 1000 had been tested, about 34% of the 481 CSF specimens would have been tested, which would have detected about 92% of the 122 specimens that were RT-PCR positive. Data were censored at a CSF WBC cut-off of 10,000. Data are for culture-negative and culture unknown specimens. Includes 188 patients with unknown culture results.

There were 162 patients who were culture positive for one of the three target organisms and 460 who met the CSF criteria (Table 1). Of the 460 patients, 123 were culture positive for one of the three target organisms and 337 were culture negative. The median age was 9 years, 60.9% were male, and 9.6% were recorded as having died. Around a third of patients had the presence of antibiotic in CSF. Among 11 of the hospitals, the proportion of patients with presence of antibiotic in CSF ranged from 0.0% to 50.0% and in the one remaining hospital, a large referral facility, the proportion was 88.1%. For some analyses (Figures 1, 2, 3), an additional 188 specimens from patients with unknown culture results were included. Among 428 CSF specimens, 173, 70, and 3 were RT-PCR positive for *N. meningitidis, S. pneumoniae,* or *H. influenzae,* respectively. For 232 serum specimens tested, 48, 17, and 0 were RT-PCR positive for the same organisms, respectively.

**Table 1:** Characteristics of patients included in the study.

Characteristic	No. (%) (n = 499)*
**Male gender**	301 (60.9%)
**Age (median, range)**	9 (0–82)
**Deceased**	40 (9.6%)
**Culture-positive**	
* N. meningitidis*	107 (21.4%)
* S. pneumoniae*	50 (10.0%)
* H. influenzae*	5 (1.0%)
**CSF ≥100 WBCs and ≥60% PMNs**	460 (93.7%)
Culture positive, 1 of 3 target organisms	123
Culture negative	337
**Positive CSF antibiotic assay**	142 (34.6%)

Of the 499 patients, 263 had CSF sample only, 67 had serum sample only, and 169 had both CSF and serum samples.

*Denominators for some characteristics <499 because of missing data for some patients

SF, cerebrospinal fluid; WBCs, white blood cells; PMNs, polymorphonuclear cells

The sensitivity of RT-PCR in CSF for diagnosis of meningitis was 100% (95% confidence limits, 96.0%–100%) for *N. meningitidis*, 97.8% (88.5%–99.9%) for *S. pneumoniae*, and 66.7% (9.4%–99.2%) for *H. influenzae* (Table 2). Specificity for the three organisms ranged from 98.9% to 100% (specific methods 1 and 2, adjusted), with narrow confidence limits. Positive and negative predictive values (PPV and NPV, respectively) of RT-PCR in CSF ranged from 98.3%–100% and 98.9%–100%, respectively (data not shown). For RT-PCR in serum, the sensitivities were lower and specificities were 94.1%–100% (Table 2).

**Table 2:** Determination of sensitivity and specificity of RT-PCR assay for *N. meningitidis* (*ctrA*), *S. pneumoniae* (*lytA*), and *H. influenzae* (*bexA*) in cerebrospinal fluid and serum specimens (see text for details).

Pathogen	No.*	Sensitivity	95% CIs	Specificity-Method 1**	No.*	95% CIs	Specificity-Method 2, unadjusted***	No.	95% CIs	Specificity-Method 2, adjusted****	No.*	95% CIs
**CEREBROSPINAL FLUID**												
***N. meningitidis***	90	100%	96.0–100	100%	51	93.0–100	75.7%	342	70.8–80.2	98.9%	262	96.7–99.8
***S. pneumoniae***	46	97.8%	88.5–99.9	100%	94	96.2–100	93.5%	386	90.6–95.8	100%	361	99.0–100
***H. influenzae***	3	66.7%	9.4–99.2	100%	139	97.4–100	99.8%	433	98.7–100	100%	432	99.2–100
**SERUM**												
***N. meningitidis***	21	57.1%	34.0–78.2	100%	12	73.5–100	83.1%	223	77.4–87.9	94.1%	188	89.8–97.0
***S. pneumoniae***	10	80.0%	44.4–97.5	100%	25	86.3–100	96.2%	235	92.9–98.2	100%	226	98.4–100
***H. influenzae***	2	0%	0–84.2	100%	33	89.4–100	100%	243	98.5–100	100%	243	98.5–100

*Number of specimens contributing to the calculation of sensitivity or specificity

**Specificity determined using specimens positive for other organisms

***Specificity determined using specimens positive for other organisms or culture negative

****Specificity determined using specimens positive for other organisms or culture negative but positive for second gene target

### Molecular genogrouping of *N. meningitidis* in CSF

Among 90 meningococcal culture-positive patients, the serogroup distribution as determined by molecular genogrouping was 10 (11.1%) group B, 67 (74.4%) group C, 11 (12.2%) group W-135, 1 (1.1%) group Y, and 1 (1.1%) non-groupable. For 83 culture negative, RT-PCR positive cases, the distribution was 10 (12.0%) group B, 64 (77.1%) group C, 4 (4.8) group W-135, 1 group Y (1.2%), and 4 (4.8%) non-groupable.

### Increased yield of RT-PCR over and above culture-based results

A total of 26 culture-negative, RT-PCR-positive patients with pneumococcal meningitis were identified, for additional yield of 52.0% of RT-PCR over the 50 culture-based results; of the 26, 23 were identified by testing CSF only, 2 by both CSF and serum, and 1 by serum only. For *N. meningitidis*, there were 91 culture negative patients with positive RT-PCR results for an additional yield of RT-PCR over the 107 culture-positive patients, an 85.0% increased yield; of the 91, 67 were identified by testing CSF only, 13 by both CSF and serum and 11 by serum only. The increased yield was 20% for *H. influenzae*, with 5 culture positive cases and 1 additional RT-PCR, culture negative case, which was identified by testing CSF.

### Relationship between CSF characteristics and RT-PCR positivity

Among culture-negative and culture-unknown specimens, there was a strong relationship between the WBC and percent neutrophils in CSF and the percent of CSFs that were RT-PCR positive (Figures 1 and 2). For example, 4.9% of CSF specimens with 0–499 WBCs were positive, as compared to 73.2% for CSFs with at least 2,000 WBCs (Figure 1). Similarly, the proportion of specimens that were RT-PCR positive increased with increasing CSF percent neutrophils (Figure 2).

An analysis of all culture negative and culture unknown CSF specimens that were tested was conducted to determine optimal CSF cut-offs (Figure 3). For example, if we had used a cut-off of at least 500 CSF leukocytes/mm^3^ to determine which specimens should undergo RT-PCR testing, we would have tested only 44% of submitted specimens yet identified 95% of specimens that were RT-PCR positive. Alternatively, a cut-off of at least 1,000 CSF leukocytes/mm^3^would have required the testing of only 34% of specimens, which would have resulted in detection of 92% of positives.

### Risk factors for being culture negative and RT-PCR positive

In univariate analysis of risk factors for being a culture negative, RT-PCR positive case (n = 118), using culture positive patients as controls (n = 162), presence of antibiotic in CSF (odds ratio 15.5, 95% CL 8.3-29.1, p<0.0001), age ≥18 (OR 2.3, 95% CL 1.4, 3.7, p<0.0012), higher CSF WBC (median 4,400 among cases, 1,280 among controls, p<0.0001), and being from one of three hospitals (hospital numbers 3, 6, or 11) (OR 6.8, 95% CL 4.0-11.6) were all associated with being a case (Table 3).

**Table 3:** Univariate analysis of risk factors for being a RT-PCR positive, culture-negative case-patient, using culture positive patients as controls.

Risk Factor	Cases, No. (%)	Controls, No. (%)	OR	95% CI	p-value
**Male gender**	69 (58.5%)	102 (63.4%)	0.8	0.5-1.3	0.41
**Age ≥18 years**	59 (50.0%)	49 (30.6%)	2.3	1.4–3.7	0.0012
**Hospitalized**	113 (99.1%)	160 (99.4%)	0.7	0.04–11.4	0.81
**Race - White**	61 (67.8%)	66 (55.9%)	Baseline		0.22
** White vs. Black**	4 (4.4%)	7 (5.9%)	0.6	0.2–2.2	
** White vs. Brown**	25 (27.8%)	45 (38.1%)	0.6	0.3–1.1	
**Antibiotic in CSF**	79 (76.7%)	25 (17.5%)	15.5	8.3–29.1	<0.0001
**CSF MIC (mm)**	21.0 (7–53)	21.0 (9–40)			0.59
**CSF glucose**	16 (0–101)	10 (0–610)			0.06
**CSF WBC count (cell/mm^3^)**	4,400 (100–29600)	1,280 (1–95200)			<0.0001
**CSF % neutrophils**	89 (60–99)	86 (0–100)			0.11
**CSF protein (xx)**	286.5 (22–4854)	230 (11–2789)			0.14
**Hospital**					
** Hospital 10**	1 (0.9%)	22 (13.6%)	Baseline		<0.0001
** Hospital 1 vs. 10**	10 (8.5%)	22 (13.6%)	10.0	1.2–84.9	
** Hospital 2 vs. 10**	8 (6.8%)	21 (13.0%)	8.4	0.96–72.9	
** Hospital 3 vs. 10**	19 (16.1%)	26 (16.1%)	16.1	2.0–129.9	
** Hospital 4 vs. 10**	1 (0.9%)	2 (1.2%)	11.0	0.5–250.9	
** Hospital 5 vs. 10**	5 (4.2%)	22 (13.6%)	5.0	0.5–46.4	
** Hospital 6 vs. 10**	8 (6.8%)	4 (2.5%)	44.0	4.3–454.9	
** Hospital 7 vs. 10**	2 (1.7%)	5 (3.1%)	8.8	0.7–117.2	
** Hospital 8 vs. 10**	2 (1.7%)	14 (8.6%)	3.1	0.3–38.0	
** Hospital 9 vs. 10**	4 (3.4%)	9 (5.6%)	9.8	0.96–99.9	
** Hospital 11 vs. 10**	57 (48.3%)	13 (8.0%)	96.5	11.9–781.9	
** Hospital 12 vs. 10**	1 (0.9%)	2 (1.2%)	11.0	0.5–250.9	
**Hospital 3, 6, 11 vs. others**	84 (71.2%)	43 (26.5%)	6.8	4.0–11.6	<0.0001
**Bacterial species**					
*** N. meningitidis***	91 (77.1%)	108 (66.7%)	1.7	0.98–2.9	0.06
*** S. pneumoniae***	26 (22.0%)	50 (30.9%)	0.6	0.4–1.1	0.10
*** H. influenzae***	1 (0.9%)	5 (3.1%)	0.3	0.03–2.3	0.23

There were a total of 118 case-patients and 162 controls. For some calculations, denominators differ because of missing data for some patients.

OR, odds ratio; CI, confidence interval; CSF, cerebrospinal fluid; MIC, minimum inhibitory concentration of CSF against strain of *Kocuria rhizophila* among specimens with detectable antibiotic; WBC, white blood cell.

In multivariable analysis, being from one of the three hospitals, presence of antibiotic in CSF, age ≥18, and infection with *N. meningitidis* were independent risk factors (Table 4). Hospitals 3, 6, and 11 had the highest proportion of cases with antibiotic in CSF (35.7%, 41.7%, and 88.1%, respectively).

**Table 4:** Multivariable analysis of risk factors for being a RT-PCR positive, culture-negative case-patient, using culture positive patients as controls.

Risk Factor	OR	95% CI	p-value
**Hospital 3, 6, or 11**	4.3	2.1–8.6	<0.0001
**Antibiotic in CSF**	12.2	5.9–25.0	<0.0001
**Age ≥18 years**	2.8	1.3–5.8	0.006
***N. meningitidis***	3.3	1.5–7.7	0.005

There were a total of 103 case-patients and 142 controls.

OR, odds ratio; CI, confidence interval; CSF, cerebrospinal fluid.

## Discussion

We found that RT-PCR, when incorporated into routine public health surveillance performed well and added substantially to estimates of public health burden for *N. meningitidis* and *S. pneumoniae.* This indicates that RT-PCR could be used as an adjunct for surveillance for bacterial meningitis in developing countries. Although Brazil is a middle-income country, RT-PCR has been demonstrated to be useful in all types of settings, from low income to highly developed countries [4], [6], [7]. RT-PCR has the added advantage of providing results more rapidly than culture and has also been shown to be an excellent tool for epidemics [15]. We performed RT-PCR in a public health reference laboratory to increase the sensitivity of bacterial meningitis surveillance, not for clinical diagnosis. However, when used in the clinical setting, timeliness is a major advantage of RT-PCR because it can generally be completed on the same day as opposed to bacterial culture, which generally requires 2–3 days.

The RT-PCR assay we used had a high sensitivity and specificity in CSF when compared to bacterial culture. A high sensitivity (around 90%) has been previously described for RT-PCR in CSF for *N. meningitidis* and *S. pneumoniae* [4]. We found that the RT-PCR assay we used, although quite specific, was not as sensitive in serum, which is in line with a recent systematic review and meta-analysis [16]. Serum is known to contain PCR inhibitors and the DNA polymerase we used is sensitive to inhibition [17], [18]. In addition, collection of blood in ethylenediaminetetraacetic acid (EDTA) is generally preferred over serum for PCR because of EDTA inhibition of DNA degradation. Despite the relative lack of sensitivity, RT-PCR in serum still managed to detect a substantial number of culture-negative cases. However, given the high sensitivity of the assay in CSF and the modest increased yield from testing serum alone, one could argue that serum should be tested only if a CSF specimen were not available.

Lack of specificity of PCR for detection of *S. pneumoniae* in serum using *ply* as the gene target in children with high rates of pneumococcal carriage has been reported [19]. However, the specificity of the pneumococcal component of our assay in serum was very high, despite inclusion of sera from 31 children <2 years old (data not shown) in the specificity calculation; this may be due to the higher specificity of *lytA* as a primary gene target as compared to *ply* [20]. The small number of *H. influenzae* cases was due to the decline in Hib disease following incorporation of Hib conjugate vaccines into the routine pediatric immunization schedule in Brazil, which limited our ability at assess performance of the *H influenzae* component of our assay.

This study points out the difficulty in evaluating a diagnostic test that performs better than the “gold standard”, bacterial culture in this case, to which it is compared. Culture of normally sterile body fluids is generally considered to be very specific but can suffer from lack of sensitivity, particularly in the presence of antibiotics. This phenomenon can make the diagnostic test appear to be less specific than it truly is when culture negative specimens (some of which may have the presence of the organism despite the negative culture) are included. We attempted to circumvent this problem by measuring specificity first by using only specimens that were culture positive for another pathogen and second after correcting for what appeared to be false positive RT-PCR results by testing these “false positives” using a second gene target. In this study, the calculated specificities were very high using both approaches, which makes us confident that we have accurately assessed the true specificity of the assay.

The results of this study suggest that surveillance programs that use RT-PCR for diagnosis of bacterial meningitis can establish WBC count cut-offs that optimize both detection of cases and utilization of laboratory resources. For example, a large number of samples could be excluded without missing a substantial proportion of bacterial meningitis cases using WBC count cut-offs of, for example, 500 or 1000. Programs with patient characteristics that differ from those in our study should consider similar analyses to determine the optimal cut-offs for their setting.

We were able to determine the meningococcal group among culture-negative cases using a PCR-based assay; a similar approach is available for *H. influenzae* (10, 12). We are also exploring non-culture approaches for determining pneumococcal serotype [21]. These serotype and serogroup data are important for monitoring the impact of the conjugate vaccines that are currently being used in Brazil.

We found that the most important risk factor for being culture negative/RT-PCR positive was presence of antibiotic in CSF, which has been previously described [6]. This finding is not surprising because antibiotics are widely available over-the-counter in Brazil [22]. In addition, it is likely that some patients were given antibiotics by healthcare workers before CSF and/or blood were obtained for culture. We suspect that the finding that being from one of three hospitals was a risk factor for being culture negative, RT-PCR positive was due to residual confounding because these hospitals had the highest rates of CSF specimens with the presence of antibiotic. However, other hospital-specific factors cannot be excluded, such as how specimens were collected, stored, and prepared for submission at the hospitals. The finding that infection with *N. meningitidis* is a risk factor for being culture-negative, RT-PCR positive may reflect the fact that this organism tends to be sensitive to most antibiotics, whereas drug resistance is common among *S. pneumoniae* isolates submitted to IAL [23], [24]. Why age ≥18 years was a risk factor is unclear but residual confounding may be a factor.

Public health surveillance in Brazil has been changed substantially as a result of this study. RT-PCR is now performed routinely on all serum and CSF specimens from patients with suspected bacterial meningitis submitted to IAL. In addition, IAL is acquiring two additional RT-PCR systems for its regional laboratories to expand availability of the assay in São Paulo State. Furthermore, the Brazilian Ministry of Health is asking all state reference laboratories to introduce RT-PCR into their routine.

In conclusion, RT-PCR for diagnosis of bacterial meningitis was successfully incorporated into public health surveillance for bacterial meningitis in São Paulo. Future studies will involve use of novel molecular approaches that could supplant our current assay and provide diagnosis for a broader array of pathogens [25], [26]. Despite success of this program in increasing the proportion of laboratory diagnosed bacterial meningitis cases, we still have a substantial number of pyogenic meningitis cases without an etiologic diagnosis. Given the public health impact and potential vaccine preventability of this disease, further investigation is needed.

## References

[1] Adams WG, Deaver KA, Cochi SL, Plikaytis BD, Zell ER (1993). Decline of childhood *Haemophilus influenzae* type b (Hib) disease in the Hib vaccine era.. JAMA.

[2] Whitney CG, Farley MM, Hadler J, Harrison LH, Bennett NM (2003). Decline in invasive pneumococcal disease after the introduction of protein-polysaccharide conjugate vaccine.. N Engl J Med.

[3] Hsu HE, Shutt KA, Moore MR, Beall BW, Bennett NM (2009). Effect of pneumococcal conjugate vaccine on pneumococcal meningitis.. N Engl J Med.

[4] Corless CE, Guiver M, Borrow R, Edwards-Jones V, Fox AJ (2001). Simultaneous detection of *Neisseria meningitidis, Haemophilus influenzae,* and *Streptococcus pneumoniae* in suspected cases of meningitis and septicemia using real-time PCR.. J Clin Microbiol.

[5] Kilpatrick ME, Mikhail IA, Girgis NI (1987). Negative cultures of cerebrospinal fluid in partially treated bacterial meningitis.. Trop Geogr Med.

[6] Saha SK, Darmstadt GL, Yamanaka N, Billal DS, Nasreen T (2005). Rapid diagnosis of pneumococcal meningitis: implications for treatment and measuring disease burden.. Pediatr Infect Dis J.

[7] Chanteau S, Sidikou F, Djibo S, Moussa A, Mindadou H (2006). Scaling up of PCR-based surveillance of bacterial meningitis in the African meningitis belt: indisputable benefits of multiplex PCR assay in Niger.. Trans R Soc Trop Med Hyg.

[8] Afifi S, Wasfy MO, Azab MA, Youssef FG, Pimentel G (2007). Laboratory-based surveillance of patients with bacterial meningitis in Egypt (1998-2004).. Eur J Clin Microbiol Infect Dis.

[9] Pedro LG, Boente RF, Madureira DJ, Matos JA, Rebelo CM (2007). Diagnosis of meningococcal meningitis in Brazil by use of PCR.. Scand J Infect Dis.

[10] Wang X, Mair R, Hatcher C, Theodore MJ, Edmond K (2011). Detection of bacterial pathogens in Mongolia meningitis surveillance with a new real-time PCR assay to detect *Haemophilus influenzae*.. Int J Med Microbiol.

[11] Mothershed EA, Sacchi CT, Whitney AM, Barnett GA, Ajello GW (2004). Use of real-time PCR to resolve slide agglutination discrepancies in serogroup identification of *Neisseria meningitidis*.. J Clin Microbiol.

[12] Carvalho MD, Tondella ML, McCaustland K, Weidlich L, McGee L (2007). Evaluation and improvement of real-time PCR detection assays to *lytA*, *ply*, and *psaA* genes for detection of pneumococcal DNA.. J Clin Microbiol.

[13] Maaroufi Y, De Bruyne JM, Heymans C, Crokaert F (2007). Real-time PCR for determining capsular serotypes of *Haemophilus influenzae*.. J Clin Microbiol.

[14] Markowitz M, Gordis L (1968). A mail-in technique for detecting penicillin in urine: application to the study of maintenance of prophylaxis in rheumatic fever patients.. Pediatrics.

[15] Nathan N, Rose AM, Legros D, Tiendrebeogo SR, Bachy C (2007). Meningitis serogroup W135 outbreak, Burkina Faso, 2002.. Emerg Infect Dis.

[16] Avni T, Mansur N, Leibovici L, Paul M (2010). PCR using blood for diagnosis of invasive pneumococcal disease: systematic review and meta-analysis.. J Clin Microbiol.

[17] Radstrom P, Knutsson R, Wolffs P, Lovenklev M, Lofstrom C (2004). Pre-PCR processing: strategies to generate PCR-compatible samples.. Mol Biotechnol.

[18] Abu Al-Soud W, Radstrom P (1998). Capacity of nine thermostable DNA polymerases To mediate DNA amplification in the presence of PCR-inhibiting samples.. Appl Environ Microbiol.

[19] Dagan R, Shriker O, Hazan I, Leibovitz E, Greenberg D (1998). Prospective study to determine clinical relevance of detection of pneumococcal DNA in sera of children by PCR.. J Clin Microbiol.

[20] Abdeldaim G, Herrmann B, Molling P, Holmberg H, Blomberg J (2010). Usefulness of real-time PCR for *lytA*, *ply*, and *Spn9802* on plasma samples for the diagnosis of pneumococcal pneumonia.. Clin Microbiol Infect.

[21] Findlow H, Laher G, Balmer P, Broughton C, Carrol ED (2009). Competitive inhibition flow analysis assay for the non-culture-based detection and serotyping of pneumococcal capsular polysaccharide.. Clin Vaccine Immunol.

[22] Silva LR, Vieira EM (2004). [Pharmacists' knowledge of sanitary legislation and professional regulations].. Rev Saúde Pública.

[23] Mantese OC, de Paula A, Almeida VV, de Aguiar PA, Wolkers PC (2009). Prevalence of serotypes and antimicrobial resistance of invasive strains of pneumococcus in children: analysis of 9 years.. J Pediatr (Rio J).

[24] Brandileone MC, Casagrande ST, Guerra ML, Zanella RC, de Andrade AL (2005). Increase in penicillin resistance of invasive *Streptococcus pneumoniae* in Brazil after 1999.. J Antimicrob Chemother.

[25] Boving MK, Pedersen LN, Moller JK (2009). Eight-plex PCR and liquid-array detection of bacterial and viral pathogens in cerebrospinal fluid from patients with suspected meningitis.. J Clin Microbiol.

[26] Ecker DJ, Sampath R, Massire C, Blyn LB, Hall TA (2008). Ibis T5000: a universal biosensor approach for microbiology.. Nat Rev Microbiol.

